# Prognostic significance of CXCR7 in cancer patients: a meta-analysis

**DOI:** 10.1186/s12935-018-0702-0

**Published:** 2018-12-19

**Authors:** Huiqian Fan, Weijun Wang, Jingjing Yan, Li Xiao, Ling Yang

**Affiliations:** Division of Gastroenterology, Union Hospital, Tongji Medical College, Huazhong University of Science and Technology, Wuhan, China

**Keywords:** CXCR7, Cancer, Prognosis, Meta-analysis

## Abstract

**Background:**

CXC chemokine receptor 7 (CXCR7) is frequently overexpressed in a variety of tumors. Nevertheless, whether CXCR7 can be used as a tumor prognosis marker has not been systematically assessed. The current meta-analysis was performed to obtain an accurate evaluation of the relationship between CXCR7 level and the prognosis of cancer patients.

**Methods:**

Embase, Web of Science, and PubMed were systematically searched according to a defined search strategy up to June 11, 2018. Then, the required data were extracted from all qualified studies which were screened out based on the defined inclusion and exclusion criteria. Finally, the hazard ratios (HR) with 95% confidence intervals (CI) were used to evaluate the prognostic significance of CXCR7 in tumor patients.

**Results:**

A total of 28 original research studies comprising 33 cohorts and 5685 patients were included in this meta-analysis. The results showed that CXCR7 overexpression was significantly related to worse overall survival (OS) (HR 1.72; 95% CI 1.49–1.99), disease-free survival (DFS) (HR 5.58; 95% CI 3.16–9.85), progression-free survival (PFS) (HR 2.83; 95% CI 1.66–4.85) and recurrence-free survival (RFS) (HR 1.58; 95% CI 1.34–1.88) in cancer patients. Furthermore, for certain types of cancer, significant associations between higher CXCR7 expression and worse OS of glioma (HR 1.77; 95% CI 1.43–2.19), breast cancer (HR 1.45; 95% CI 1.28–1.63), esophageal cancer (HR 2.72; 95% CI 1.11–6.66) and pancreatic cancer (HR 1.46; 95% CI 1.12–1.90) were found. However, for lung cancer and hepatocellular cancer, there was no significant relationship between CXCR7 expression level and OS, (HR 2.40; 95% CI 0.34–17.07) and (HR 1.37; 95% CI 0.84–2.24) respectively.

**Conclusions:**

Increased CXCR7 level could predict poor prognosis of tumor patients and might be regarded as a novel prognostic biomarker for tumor patients.

## Background

Cancer is the primary cause of death in both developing and developed countries. In 2018, an estimated number of 1,735,350 new cancer cases and 609,640 cancer deaths are predicted to happen in the United States [1]. Due to the growth and aging of the population, as well as the increasing prevalence of established cancer risk factors, including overweight, physical inactivity, smoking, and changing reproductive patterns, the burden of cancer is still growing worldwide [2]. Although there are plenty of treatments for cancer, considering the low efficacy of treatments and poor prognosis of tumor patients, targeted therapies are desperately needed. Numerous biomarkers have been explored to improve the efficacy of oncotherapy and predict the prognosis of cancer patients. However, most cancer biomarkers currently used are not satisfactory [3]. Hence, it is essential to develop novel cancer biomarkers, not only provide novel therapeutic targets but also improve prognosis.

Chemokines are small proteins that primarily regulate cell trafficking and the differentiation and functions of various tissues [4]. Furthermore, chemokines and their receptors have been regarded as mediators of chronic inflammation, which exerts considerable influence on the development and progression of tumors [5]. Accumulating evidence has indicated that chemokines play crucial roles in the tumorigenesis and progress of cancer [6]. Among various chemokines, CXCL12 and CXCR4 are the most thoroughly investigated molecules. CXCR4 is the first identified receptor for CXCL12, and it is generally recognized that the CXCL12/CXCR4 axis participates in many aspects of cancer, such as the angiogenesis, metastasis, and the survival of cancer cells [7]. Consistent with the important roles of CXCL12/CXCR4 in cancer, many studies have proven that high levels of CXCL12 and CXCR4 are related to worse prognosis in various malignant tumors [8, 9].

CXC chemokine receptor 7 (CXCR7) is a newly found receptor for CXCL12, which is an atypical, chemokine-specific seven-transmembrane G protein-coupled receptor that does not mediate typical chemokine responses such as modulation of intracellular calcium mobilization or adenylyl cyclase activity [10]. Studies have demonstrated that the binding affinity of CXCL12 to CXCR7 is tenfold higher than to CXCR4. Similar to CXCR4, CXCR7 can also serve as a crucial regulator in several physiologic processes [11]. CXCR7 exerts essential functions in embryonic development and takes part in weakening chemotaxis of T lymphocytes induced by CXCL12 [11–14]. It also participates in trafficking of germ and progenitor cells during tissue repair and development [15, 16].

Recent studies have also reported that CXCR7 participates in tumorigenesis and tumor progress. Emerging evidence suggests that CXCR7 is extensively expressed in various tumor tissues and has the function of activating endothelial cells [17, 18], promoting the proliferation, migration, invasion, and metastasis of cancer cells [19–24]. In bladder cancer, high expression of CXCR7 has been associated with the proliferation, migration, and invasion of cancer cells, leading to rapid tumor progress [25]. Moreover, high expression of CXCR7 has indicated more lymphovascular invasion, regional lymph node metastasis and severe invasion in extramammary Paget disease [26]. Nowadays, some people have proposed that CXCR7 could be a novel prognostic biomarker for cancer patients. But because most studies published currently have limitation of sample size and discrete outcome, there is insufficient evidence to confirm the relationship between CXCR7 expression and the prognosis of cancer patients. Therefore, the present meta-analysis was performed to systematically evaluate the prognostic significance of CXCR7 expression in tumor patients.

## Materials and methods

### Study strategy

This meta-analysis was performed according to the preferred reporting items for systematic reviews and meta-analyses (PRISMA) statement [27]. For this study, we retrieved publications before 11 June 2018 from Embase, Web of Science, and PubMed using the following search terms: “CXCR7 or CXC chemokine receptor 7 or RDC1 or ACKR3 or GPRN1” AND “neoplasm or tumor or cancer or malignancy or carcinoma” AND “survival or prognosis or outcome or prognostic”. All relevant publications in reference lists were also searched manually to identify potentially qualified papers.

### Inclusion and exclusion criteria

In the present meta-analysis, the eligible studies or cohorts must have meet the following criteria: (1) The expression level of CXCR7 was detected in human tissues or plasma samples; (2) Tumors were diagnosed accurately by histopathology; (3) The relationship between CXCR7 expression level and survival rates of patients was evaluated; (4) Studies provided sufficient information to calculate the hazard ratio (HR) for survival rates, with 95% confidence intervals (CI). Studies were excluded if they met any of the following criteria: (1) duplicate publications; (2) conference abstracts, case reports, reviews, patents, letters, non-English or unpublished articles; (3) studies merely concerned with cancer cell lines or animal models; (4) HRs and 95% CIs could not be extracted or calculated due to insufficient data.

### Data extraction

Two researchers independently extracted all necessary data and reached an agreement on all contents. The third author made any decisions about confusing items. The extracted information in each study included: the first author’s name, the year of publication, region of the population enrolled, tumor type, sample size (high/low), follow-up time, type of sample detected, the endpoints, high or low expression accounting for poor prognosis, cut-off value, methods of obtaining HRs (directly or indirectly), survival analysis method and Newcastle–Ottawa Scale (NOS) score. Overall survival (OS), disease-free survival (DFS), progression-free survival (PFS) and recurrence-free survival (RFS) were considered to be endpoints. HRs that were directly obtained or calculated from Kaplan–Meier curves served as parameters to evaluate the relationship between CXCR7 expression level and prognosis of cancer patients [28].

### Quality assessment

The NOS score ranging from 0 to 9 was used to assess the quality of the cohort studies. This system included the following three categories to evaluate each study: selection of study groups, comparability of groups and ascertainment of outcomes. A NOS scores ≥ 7 indicated high quality, and a NOS scores < 7 indicated low quality.

### Statistical analysis

Pooled HRs (high/low) and their associated 95% CIs which were calculated using Stata version 14.0 (Stata Corporation, College Station, TX, USA) demonstrated the relationship between CXCR7 expression level and prognosis of tumor patients. The heterogeneity among studies was estimated by the Cochrane’s Q test and the Higgins I^2^ statistic (p < 0.10 or I^2^ > 50% was considered significant) [29]. When heterogeneity was not significant (p > 0.10 and I^2^ < 50%), the fixed-effect model was used for analysis. Otherwise, the random-effect model seemed to be more appropriate. Furthermore, meta-regression and subgroup analyses were conducted to identify potential sources of heterogeneity, and the included cohorts were divided into two subgroups based on similar characteristics. Sensitivity analysis was performed by removing each cohort sequentially to explore possible explanations for heterogeneity. Publication bias was evaluated using Begg’s test and Egger’s test [30]. Due to the publication bias in this meta-analysis, we also conducted trim and fill analysis.

## Results

### Search results

**Fig. 1:**
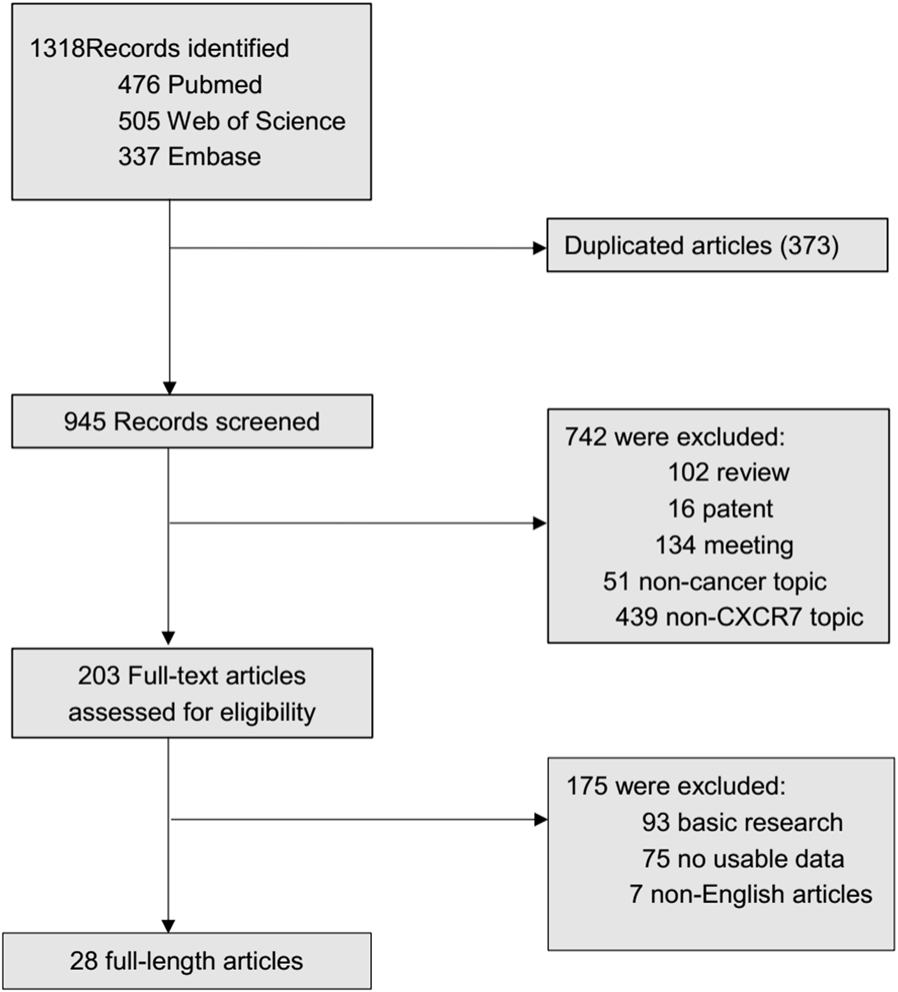
The flow diagram indicated the process of study selection

As shown in Fig. 1, a total of 1318 articles were obtained from Embase, PubMed and Web of Science utilizing the search strategy described above. First, 373 duplicate reports were removed. Subsequently, 134 meeting abstracts, 16 patents, 7 non-English articles, 102 reviews, 439 studies focusing on non-CXCR7 topics and 51 studies about noncancer topics were excluded by skimming titles and abstracts. Furthermore, we ruled out 93 basic-research studies and 75 studies lacking relevant data through close reading. Ultimately, 28 studies were included and the relevant data were extracted. Detailed information of these qualified articles is presented in Table 1. The total number of studies was 28 comprising 33 cohorts and 5685 patients in this current meta-analysis. In summary, the sample size of all eligible studies was between 30 and 840 and the follow-up time ranged from 26 to 266 months. Among all the cohorts, China (n = 13) was the most common region of studies, followed by Italy (n = 6), USA (n = 4), Japan (n = 3) Germany (n = 3), UK (n = 2), Singapore (n = 1), and Netherlands (n = 1). In terms of disease outcomes, 24 cohorts reported OS, 4 cohorts reported DFS, 8 cohorts reported RFS and 3 cohorts reported PFS. To assess the expression level of CXCR7, most studies used immunochemistry (IHC). Other methods such as RT-PCR, mRNA microarray, and cDNA-array were also applied. The types of cancer evaluated in the current meta-analysis were glioma (n = 3) [31–33], thyroid carcinoma (n = 1) [34], esophageal cancer (n = 3) [35–37], breast cancer (n = 4) [38–41], hepatocellular carcinoma (n = 2) [42, 43], lung cancer (n = 2) [44, 45], pancreatic cancer (n = 2) [23, 46], renal cancer (n = 2) [10, 47], oral carcinoma (n = 1) [48], chondrosarcoma (n = 1) [49], gastric cancer (n = 1) [50], colorectal carcinoma (n = 3) [24, 51, 52], gallbladder cancer (n = 1) [53], extramammary Paget disease (n = 1) [26] and cervical cancer (n = 1) [54].

**Table 1:** Characteristics of studies included in the meta-analysis

Author	Year	Region	Type of cancer	Sample size (high/low)	Follow-up (month)	Endpoints	Expression associated with poor prognosis	Assay method	Cut-off value	Survival analysis	NOS score	Method
Salazar et al. [31]	2018	USA	Glioma	77	113	OS	High	mRNA microarray data	Median	NA	6	1
				49	80	OS	High	mRNA microarray data	Median	NA	6	1
Werner et al. [34]	2018	Germany	Thyroid carcinoma	25/15	197	OS, RFS	High	Tissue microarray IHC	Median	Univariate multivariate	8	1
Qiao et al. [35]	2018	China	Esophageal squamous cell carcinoma	23/22	40	OS, PFS	High	IHC, RT-PCR	Median	NA	7	2
Hao et al. [39]	2018	China	Breast cancer	593	200	RFS	High	cDNA-array	NA	NA	6	1
Chang et al. [26]	2017	China	Extramammary paget disease	21/71	130	DFS	High	IHC	High: the product of intensity score and quantity score was 4–9	Univariate and multivariate	8	1
D’Alterio et al. [10]	2010	Italy	Renal cancer	100/70	180	DFS	High	IHC	High: stained (positive) tumor cells in 10 high power field (400×)/slide: > 20%.	Univariate and multivariate	8	1
Hui et al. [48]	2016	China	Oral squamous cell carcinoma	66	60	OS	High	IHC	NA	NA	6	2
Wani et al. [41]	2014	USA	Breast cancer	85/440	220	OS	High	RNA array datasets	High: ACKR3 (CXCR7 gene symbol) is greater than 1.0-fold of the standard deviation above the mean	NA	6	2
Polimeno et al. [42]	2015	Italy	Hepatocellular carcinoma	74/6	126	OS	NS	IHC	High: stained (positive) tumor cells > 60%	Univariate	7	1
				11/28	87	OS	NS	IHC	High: stained (positive) tumor cells > 60%	Univariate	7	1
D’Alterio et al. [24]	2016	Italy	Colorectal cancer	14/16	66	PFS	High	IHC	High: the rate of stained cancer cells was more than 50%	Univariate	7	1
Tachezy et al. [36]	2013	Germany	Esophageal cancer	72/87	178	OS	NS	IHC	High: spots with staining intensity ≥ 2 were classified as CXCR7 positive	NA	6	2
D’Alterio et al. [52]	2014	Italy	Rectal cancer	43/21	127	RFS	NS	IHC	High: the percentage of the total number of stained cells > 50%	Univariate	7	1
Deng et al. [32]	2017	China	Glioblastoma	77/69	43	OS	High	IHC	High: the staining intensity score of CXCR7 > 1	Univariate and multivariate	7	1
Franco et al. [44]	2012	Italy	Non-small cell lung cancer	19/26	26	OS	High	IHC	High: the percentage of the total number of stained cells > 30%	Univariate and multivariate	7	2
Gebauer et al. [46]	2011	Germany	Pancreatic adenocarcinoma	47/202	175	OS	NS	IHC	High: the percentage of the total number of stained cells > 20%	Multivariate	7	2
Goto et al. [37]	2017	Japan	Esophageal squamous cell carcinoma	40/73	97	RFS	NS	IHC	High: the expression grades in cytoplasm ≥ 1	Univariate and multivariate	7	2
Guo et al. [23]	2016	China	Pancreatic cancer	66/116	87	OS	High	IHC	High: the multiplication of positive cell proportion score and staining score was 4–9	Univariate and multivariate	7	1
				53/100	95	OS	High	IHC	High: the multiplication of positive cell proportion score and staining score was 4–9	Univariate and multivariate	7	1
Wang et al. [47]	2012	China	Renal cell carcinoma	72/25	120	OS, RFS	High	IHC	High: the percentage of the total number of stained cells ≥ 30%	Univariate and multivariate	8	1
Iwakiri et al. [45]	2009	Japan	Nonsmall cell lung cancer	44/35	66	DFS, OS	High	RT-PCR	Median	Multivariate	7	1
Li et al. [49]	2015	China	Chondrosarcoma	49/11	65	OS	High	IHC	High: the percentage of the total number of stained cells ≥ 10%	NA	7	2
Liu et al. [33]	2015	USA	Glioma	144/199	120	OS	High	Microarray	High: fourfold elevated expression of CXCR7	NA	6	2
Ribas et al. [40]	2014	UK	Breast cancer	125/375	144	RFS	High	IHC	The highest quartile of the gene expression was used to dichotomise the patients into high and low groups (> 75%)	NA	7	2
				209/631	144	RFS	High	IHC	The highest quartile of the gene expression was used to dichotomise the patients into high and low groups (> 75%)	NA	7	2
Nambara et al. [57]	2016	Singapore	Gastric Cancer	98/98	60	OS	High	RT-PCR	Median	NA	7	2
		Japan	Gastric Cancer	98/97	60	OS	High	RT-PCR	Median	NA	7	2
Schrevel et al. [54]	2012	Netherlands	Cervical cancer	43/58	266	DFS	High	IHC	High: immunoreactivity was scored as weak, moderate or strong staining intensity	Univariate and multivariate	8	1
Wu et al. [38]	2015	China	Breast cancer	57/58	89	OS	High	RT-PCR	Median	Univariate and multivariate	7	1
Xue et al. [43]	2014	China	Hepatocellular carcinoma	40/34	110	OS, RFS	High	IHC	High: the percentage of tumor cells ≥ 80% (in 2+ intensity cells) or ≥ 30% (in 3+ intensity cells) were considered high	NA	7	2
Yang et al. [51]	2015	China	Colorectal carcinoma	62/34	60	OS, PFS	High	IHC	High: the multiplication of positive cell proportion score and staining score ≥ 2	Univariate and multivariate	7	1
Yao et al. [53]	2011	China	Gallbladder cancer	49/23	95	OS	High	IHC	High: the multiplication of positive cell proportion score and staining score ≥ 2	Univariate and multivariate	7	1

*OS* overall survival, *DFS* disease-free survival, *PFS* progression-free survival, *RFS* recurrence-free survival, *IHC* immunohistochemistry, *RT-PCR* real time polymerase chain reaction, *NA* not available, *NOS* Newcastle–Ottawa Scale, *Method 1* HRs obtained directly from publications, *Method 2* HRs calculated from the total number of events, corresponding p value and data from Kaplan–Meier curves

### Relationship between CXCR7 expression level and OS of tumor patients

**Fig. 2:**
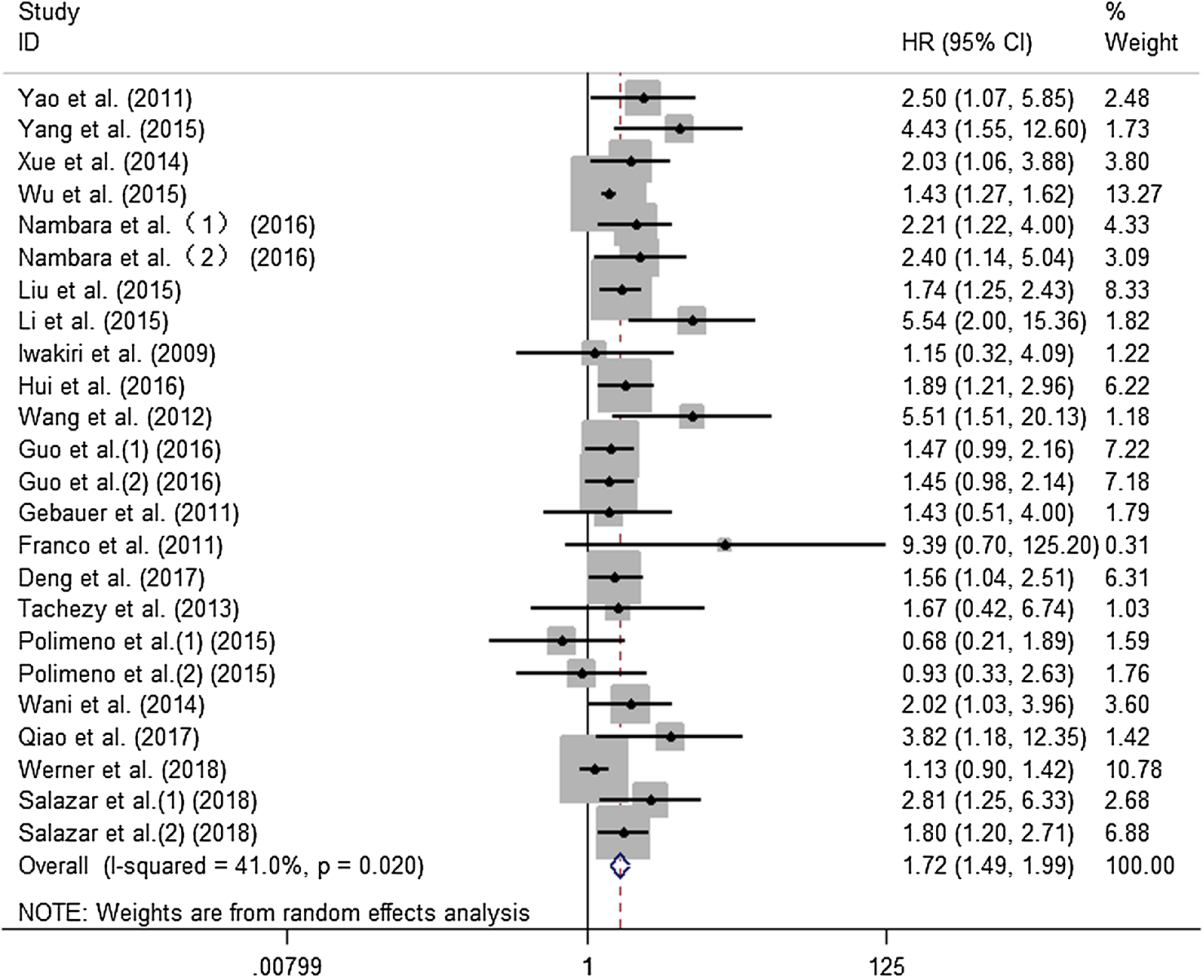
Meta-analysis of the pooled HRs of OS for cancer patients

**Fig. 3:**
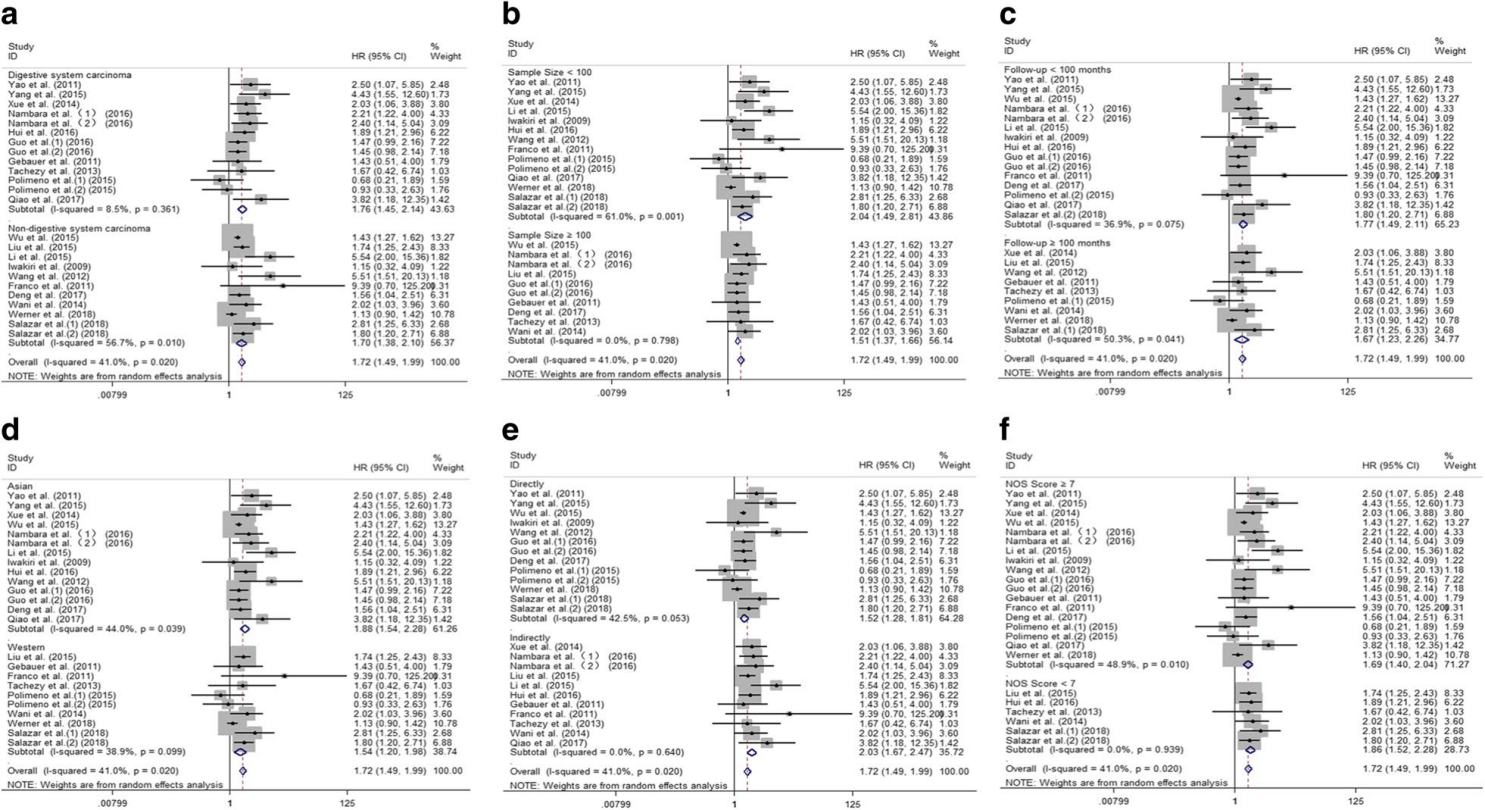
Results of subgroup analysis of pooled HRs of OS for cancer patients. **a** Subgroup analysis stratified by type of cancer. **b** Subgroup analysis stratified by sample size. **c** Subgroup analysis stratified by follow-up time. **d** Subgroup analysis stratified by the region. **e** Subgroup analysis stratified by source of HR. **f** Subgroup analysis stratified by NOS score

In total, 24 cohorts from 20 studies assessed the relationship between CXCR7 expression level and OS of cancer patients in 3182 participants. The random-effect model was used because of significant heterogeneity (I^2^ = 41.0%; p = 0.02). The pooled HR for OS of patients with high CXCR7 level compared with low expression was 1.72 (95% CI 1.49–1.99, p < 0.001), indicating that high CXCR7 level was markedly related to reduced OS of cancer patients (Fig. 2). Moreover, to explore the sources of heterogeneity, subgroup analyses according to the type of cancer (digestive system or nondigestive system carcinoma), sample size (fewer than 100 or more than 100), follow-up time (fewer than 100 or more than 100 months), region (Asia or elsewhere), methods of obtaining HRs (directly or indirectly) and paper quality (NOS scores ≥ 7 or < 7) were conducted (Fig. 3a–f). All results of subgroup analyses demonstrated significant relationship between CXCR7 overexpression and poor OS of tumor patients. We also applied meta-regression by the covariates including all mentioned factors to explore the sources of heterogeneity. However, all the above factors did not account for the sources of heterogeneity since the p values of all above factors were not less than 0.05 (Table 2). Additionally, we conducted Cox multivariate analysis from 8 studies including 9 cohorts to obtain HR. The result indicated that elevated CXCR7 expression could independently predict OS for the prognosis of tumor patients (HR 1.49, 95% CI 1.24–1.79, p < 0.001).

**Table 2:** Subgroup analysis of pooled HRs for OS in cancer patients with abnormally expressed CXCR7

Subgroup analysis	No. of cohorts	Pooled HRs	Meta regression (p-value)	Heterogeneity	
		Random		I^2^ (%)	*p*-value
Sample size			0.122		
< 100	14	2. 04 [1.49–2.81]	–	61.0	0.001
≥ 100	10	1.51 [1.3–1.66]	–	0.0	0.798
Type of cancer			0.379		
Digestive system carcinoma	13	1.76 [1.45–2.14]	–	8.5	0.361
Non-digestive system carcinoma	11	1.70 [1.38–2.10]	–	56.7	0.010
Follow-up time			0.689		
< 100	15	1.77 [1.49–2.11]	–	36.9	0.075
≥ 100	9	1.67 [1.23–2.26]	–	50.3	0.041
The region			0.229		
Asia	14	1.88 [1.54–2.28]	–	44.0	0.039
Other countries	10	1.54 [1.20–1.98]	–	38.9	0.099
NOS score			0.604		
≥ 7	18	1.69 [1.40–2.04]	–	48.9	0.010
< 7	6	1.86 [1.52–2.28]	–	0.0	0.939
Source of HR			0.074		
Directly	13	1.52 [1.28–1.81]	–	42.5	0.053
Indirectly	11	2.03 [1.67–2.47]	–	0.0	0.640

### Relationship between CXCR7 expression level and OS in certain types of cancer

**Fig. 4:**
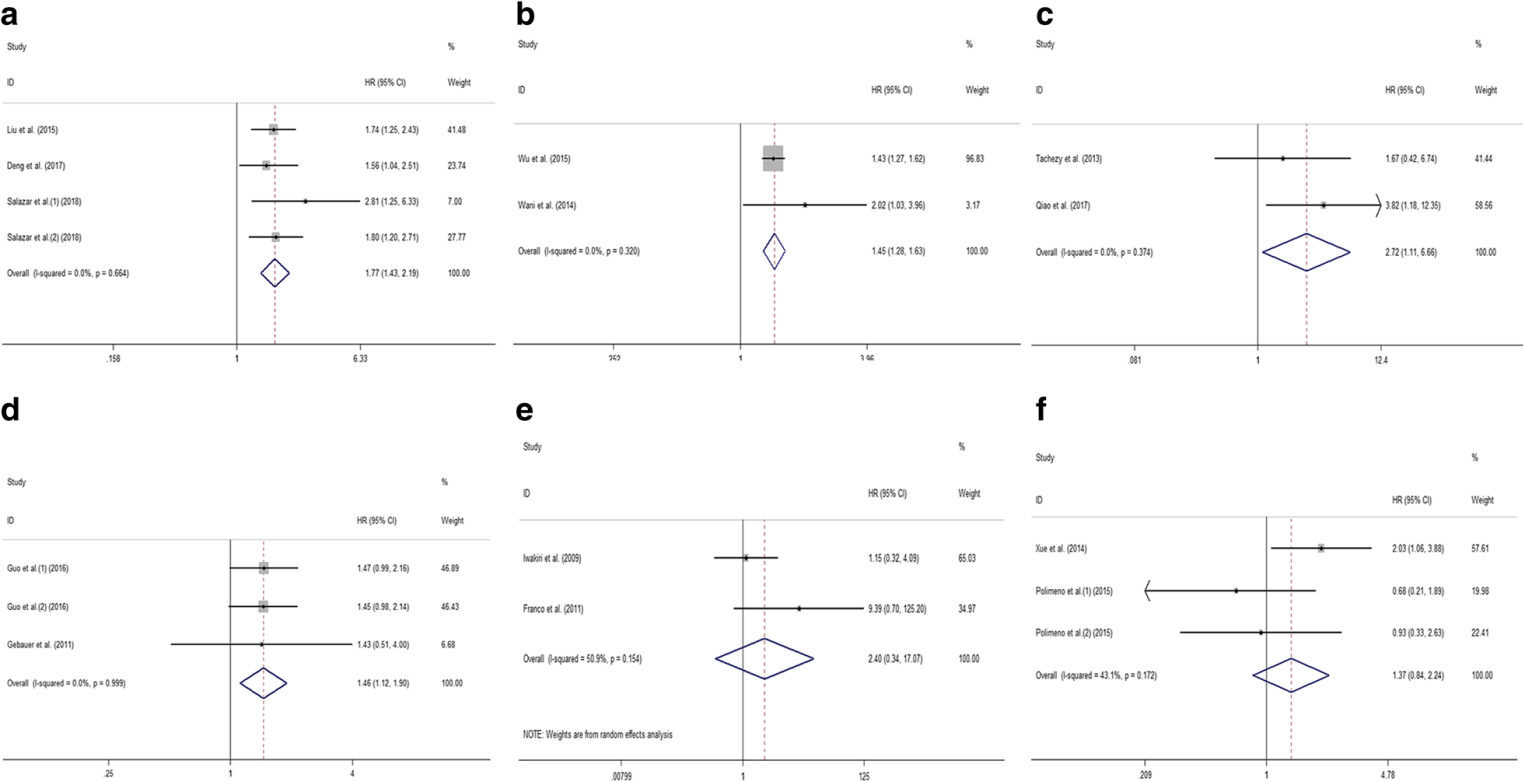
Meta-analysis of the pooled HRs of OS for glioma (**a**), breast cancer (**b**), esophageal cancer (**c**), pancreatic cancer (**d**), lung cancer (**e**) and hepatocellular cancer (**f**)

Subsequently, we explored the relationship between CXCR7 expression level and OS in certain types of tumor. Significant associations were detected between higher CXCR7 expression level and worse OS of glioma (HR 1.77; 95% CI 1.43–2.19, p < 0.001) (Fig. 4a), breast cancer (HR 1.45; 95% CI 1.28–1.63, p < 0.001) (Fig. 4b), esophageal cancer (HR 2.72; 95% CI 1.11–6.66, p = 0.029) (Fig. 4c), and pancreatic cancer (HR 1.46; 95% CI 1.12–1.90, p = 0.006) (Fig. 4d). However, for lung cancer (HR 2.40; 95% CI 0.34–17.07, p = 0.383) (Fig. 4e) and hepatocellular cancer (HR 1.37; 95% CI 0.84–2.24, p = 0.209) (Fig. 4f), no significant relationship was found between CXCR7 expression level and OS of tumor patients.

### Relationship between CXCR7 expression level and PFS, RFS and DFS of tumor patients

**Fig. 5:**
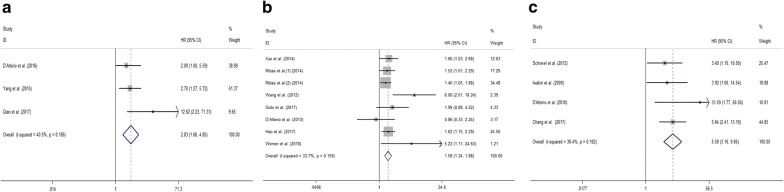
Meta-analysis of the pooled HRs of PFS (**a**), RFS (**b**) and DFS (**c**) for cancer patients

As shown in Fig. 5a, three cohorts were included in the meta-analysis of PFS. The results illuminated an obvious association between higher CXCR7 expression level and shorter PFS (HR 2.83, 95% CI 1.66–4.85, p < 0.001). Similarly, the analysis of RFS, which covered eight cohorts, showed that the tumor patients with elevated CXCR7 expression level had significantly worse RFS compared to those with lower CXCR7 (HR 1.58, 95% CI 1.34–1.88, p < 0.001) (Fig. 5b). Furthermore, an obvious association between higher CXCR7 expression level and shorter DFS was revealed by assessing four cohorts (HR 5.58; 95% CI 3.16–9.85, p < 0.001) (Fig. 5c). Considering the limited quantity of included cohorts, we did not conduct a subgroup analysis.

### Sensitivity analysis

**Fig. 6:**
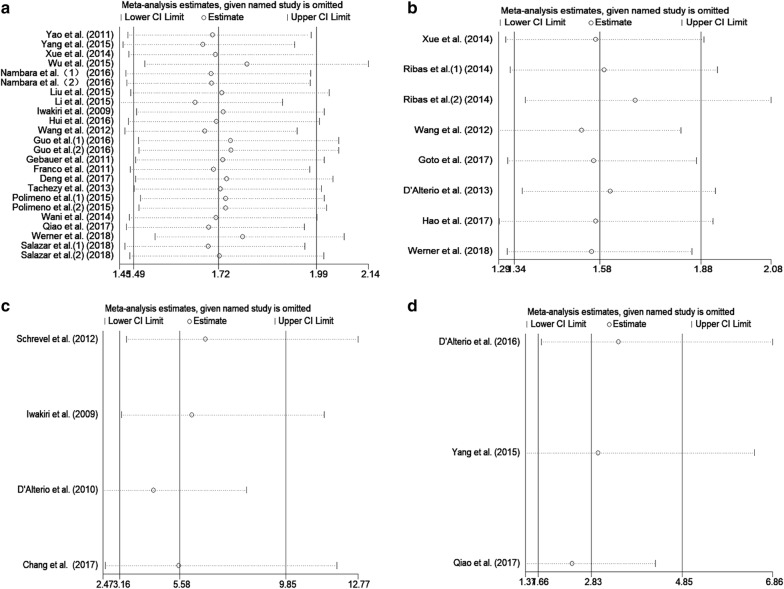
Sensitivity analysis plot of pooled HRs of OS (**a**), RFS (**b**), DFS (**c**) and PFS (**d**) for cancer patients with abnormally expressed CXCR7

We conducted sensitivity analysis to determine the influences of individual studies and the stability of our results by removing single study sequentially. For OS, the sensitivity analysis showed that the three cohorts from Wu et al., Li et al. and Werner et al. influenced the results greatly, which showed that these cohorts might account for heterogeneity. All the values of HR in the list were greater than 1, indicating that our results were stable and reliable (Fig. 6a). For RFS (Fig. 6b), the sensitivity analysis revealed that the cohort from Ribas et al. [40] affected the results greatly. In addition, all included studies had a great influence on DFS (Fig. 6c) and PFS (Fig. 6d), which indicated that the results for DFS and PFS were relatively unstable.

### Publication bias

**Fig. 7:**
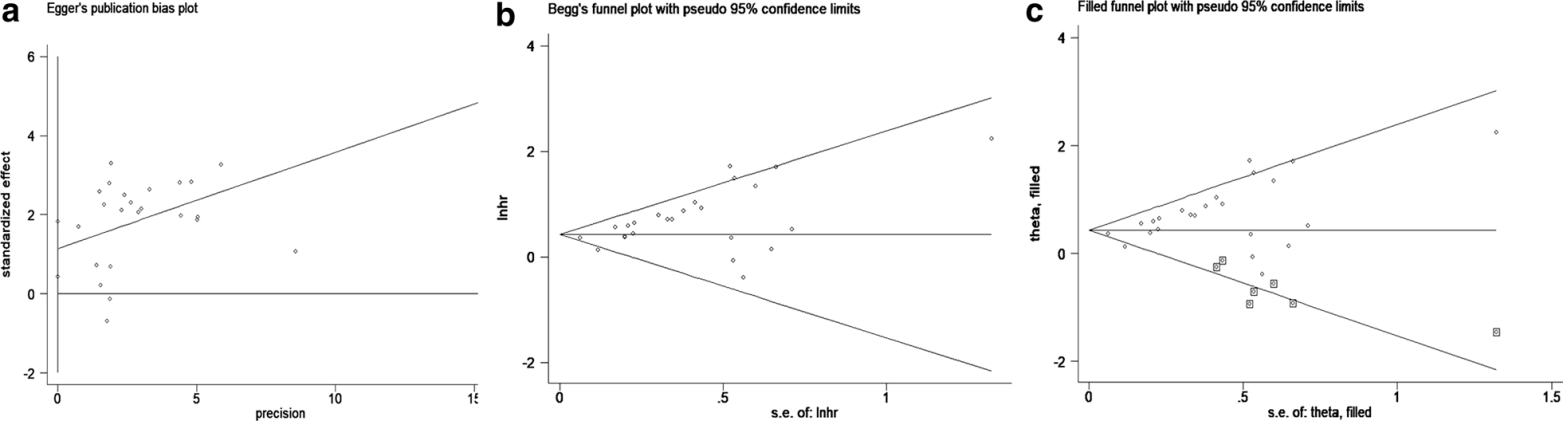
Egger’s test (**a**) and Begg’s test (**b**) for publication bias. Trim and fill analysis of the eligible studies for the present meta-analysis (**c**)

We conducted Begg’s funnel plot and Egger’s test to evaluate the publication bias. The funnel plot was obviously asymmetric. Moreover, significant publication bias (p = 0.003, p = 0.035 respectively) was identified by Egger’s linear regression test (Fig. 7a) and Begg’s test (Fig. 7b). Furthermore, we conducted the trim and fill analysis (Fig. 7c). Seven studies that focused on the value of CXCR7 expression level for predicting the OS of tumor patients remained unpublished. It is worth noting that the filled results for OS (HR = 1.54, 95% CI 1.31–1.81, p < 0.001) were in accordance with our original results.

## Discussion

So far, there is substantial evidence that CXCR7 plays a crucial part in proliferation, migration, invasion and metastasis of different cancers, indicating poor prognosis of cancer patients [41, 55]. It was reported that the expression of CXCR7 was higher in gallbladder cancer patients, which was associated with advanced TNM stage and poorer survival [53]. Gebauer et al. showed that in pancreatic adenocarcinoma, CXCR7 was highly expressed, which played important roles in tumor cell proliferation and metastasis [46]. Furthermore, the expression level of CXCR7 was significantly higher in gastric cancer cells than normal cells and elevated CXCR7 expression level was related to peritoneal metastasis and worse prognosis of gastric tumor patients [50]. Given the important functions of CXCR7 in cancer, many researchers have proposed that CXCR7 might be a potential prognostic biomarker for tumor patients [56, 57]. Nevertheless, whether CXCR7 could serve as a promising biomarker for predicting the survival of tumor patients remains controversial because most studies published to date have deficiencies in sample size and discrete outcomes.

At present, our meta-analysis systematically evaluated the reported studies concerning CXCR7 expression level and tumor patients’ prognosis. All of the survival data from 28 independent studies, including 33 cohorts and 5685 cancer patients, were systematically analyzed. The results showed that higher CXCR7 was significantly associated with worse OS of tumor patients. Because of the heterogeneity in these articles, subgroup analysis and meta-regression analysis were conducted to explore the sources of heterogeneity. The subgroup analyses indicated that the significant association between high CXCR7 expression level and poor OS of tumor patients was not changed by region, sample size, type of cancer, paper quality, source of HR or follow-up time. Moreover, the meta-regression analysis failed to identify the source of the heterogeneity in all covariates. However, by meta-regression analysis, the p-value for the source of HR was relatively low (p = 0.074), which indicated that the way we obtained HR might account for the heterogeneity of our analysis. Furthermore, we combined HRs from Cox multivariate analyses. The results demonstrated that CXCR7 acted as an independent prognostic factor for OS of tumor patients. In addition, the sensitivity analysis of OS showed that the three cohorts from Wu et al., Li et al. and Werner et al. influenced the results significantly, which showed that these three cohorts might account for the heterogeneity. All the values of HR in the list were greater than 1, indicating that our results are stable and reliable.

We also assessed the prognostic impact of CXCR7 on six kinds of cancer. The results demonstrated that higher CXCR7 expression was related to worse OS in glioma, breast cancer, esophageal cancer and pancreatic cancer, which was in accordance with previous studies. Nevertheless, for lung cancer and hepatocellular cancer, no significant association between CXCR7 expression with OS of cancer patients was found. The reason why the result of lung cancer was contrary to others might be because of the limited sample size and different clinical characteristics of recruited patients. For example, the results from Iwakiri et al. [45] have demonstrated that the significant association between high expression of CXCR7 and poor prognosis of tumor patients existed only in patients with p-stage I NSCLC, not in patients with p-stage II–III NSCLC, because tissues of p-stage II–III NSCLC include both patients exposed to preoperative therapy and not. For hepatocellular cancer, the inconsistent meta-analysis results might be the reason that CXCR7 was not the only factor influencing the prognosis of hepatocellular cancer patients. Because Polimeno et al. have found that high expression of CXCR7 was also regulated by more finely tuned CXCR4–CXCL12 level in hepatocellular cancer [42]. Thus, larger-scale, multicenter studies including all stages of patients are necessary to confirm our hypothesis for lung cancer. Furthermore, more studies are needed to explore the specific function of the CXCL12/CXCR4/CXCR7 axis in hepatocellular cancer.

The relationships between CXCR7 expression level with PFS, RFS and DFS was evaluated in our meta-analysis as well. The results indicated that cancer patients with higher CXCR7 expression level had shorter PFS, RFS and DFS. The sensitivity analysis showed that all cohorts affected the results greatly, which might be because of the small quantity of cohorts included. Thus, it will be necessary to do more research concerning the effects of CXCR7 on PFS, RFS and DFS of cancer patients.

The underlying mechanisms involved in the relationship between CXCR7 overexpression and poor prognosis of tumor patients have been extensively investigated. Accumulating evidence has proven that CXCR7 exerts pleiotropic effects in tumor cell survival, proliferation, migration, invasion and metastasis. CXCR7 can mediate epidermal growth factor receptor (EGFR) phosphorylation in a CXCR7 ligand-independent way, which enhances EGFR-mediated mitogenic signaling and plays a vital role in proliferation of prostate and breast cancer cells [58, 59]. In bone sarcomas, the binding of CXCL12 to CXCR7 can activate the PI3K-Akt-NF-κΒ and MEK-ERK-IKKαβ-NF-κΒ pathways, which regulates the proliferation/survival as well as the migration/metastasis of tumor cells [60]. Furthermore, CXCR7 might promote colorectal cancer progression via regulation of the p-ERK and β-arrestin pathways [61]. Moreover, Wu et al. [62] demonstrated that CXCR7 was responsible for TGFβ1-related cell migration, invasion, epithelial–mesenchymal transition and tumor-initiating features in lung cancer. What’s more, CXCR7/TGFβ1 coexpression was positively correlated with the expression of CD44, a cancer stem cell marker promoting lymph node metastasis in lung cancer. In addition, the entire CXCR4–CXCL12–CXCR7 axis could activate the mTOR pathway and stimulate cell migration in human A498 and SN12C renal cancer cells [63]. Thus, highly expressed CXCR7 could promote the progression of cancer via various signal pathways. However, more studies are still needed to elucidate the specific mechanisms of the pro-tumor effects of CXCR7, especially for certain types of cancer, such as hepatocellular cancer.

Some limitations existed in the present meta-analysis. First, we could not obtain HRs of some cohorts from the publications directly. The calculating method of HRs and corresponding 95% CIs through survival curves might not be precise enough. Second, all cohorts included in this meta-analysis did not agree on the cutoff value of CXCR7 expression, which could cause heterogeneity among the studies. Third, the existence of publication bias might exaggerate the influence of CXCR7 on the prognosis of cancer patients to some degree. Moreover, due to language limitations, studies published in other languages were not included because of difficulties in obtaining information accurately.

## Conclusions

The high expression of CXCR7 could act as a risk factor for shorter OS, PFS, RFS and DFS in cancer patients based on the current published data. It seems reasonable to assume that CXCR7 might become a promising biomarker for the prognosis of cancer patients. Additionally, developing strategies against CXCR7 would be a novel therapy for tumors. This meta-analysis systematically evaluated the impact of CXCR7 expression level on the prognosis of tumor patients. In the future, large-scale, well-designed studies with more information about potential correlative factors are necessary to assess the value of CXCR7 in human cancer.

## Abbreviations

CI: confidence intervals
HR: hazard ratios
OS: overall survival
DFS: disease-free survival
PFS: progression-free survival
RFS: recurrence-free survival
IHC: immunohistochemistry
RT-PCR: real time polymerase chain reaction
EGFR: epidermal growth factor receptor
